# Influence of Ethanol Grade on Captures of Ambrosia Beetles in Tree Fruit Orchards, Ornamental Nurseries, and Lumber Yards

**DOI:** 10.3390/insects15060408

**Published:** 2024-06-03

**Authors:** Marek Dzurenko, Christopher M. Ranger, Martin Pavlík, Michael E. Reding

**Affiliations:** 1 Department of Integrated Forest and Landscape Protection, Technical University in Zvolen, Ul. T. G. Masaryka 24, 960 01 Zvolen, Slovakia; pavlik@tuzvo.sk; 2 Horticultural Insects Research Laboratory, United State Department of Agriculture-Agricultural Research Service, 1680 Madison Ave., Wooster, OH 44691, USA; christopher.ranger@usda.gov (C.M.R.); mike.reding@usda.gov (M.E.R.)

**Keywords:** Scolytinae, Xyleborini, invasive species, ethanol, monitoring, artificial ecosystems

## Abstract

**Simple Summary:**

In our study, we explored ways to effectively trap invasive ambrosia beetles, which pose significant threats to trees and forests. We focused on comparing two types of bait—pure ethanol and denatured ethanol—to attract these pests in different environments like tree nurseries and orchards. We found that traps using pure ethanol captured more *Xylosandrus germanus* beetles compared to those with denatured ethanol. Our findings suggest that using pure ethanol could be more effective for monitoring certain ambrosia beetle species. This research contributes to better pest management strategies, helping protect trees from invasive pests, ultimately benefiting society by safeguarding ecosystems and agricultural productivity.

**Abstract:**

Ambrosia beetles, particularly invasive species within the tribe Xyleborini, such as *Xylosandrus germanus* (Blandford, 1894), pose significant threats to various ecosystems and managed habitats worldwide. Monitoring these invaders is vital for effective pest management, typically accomplished through ethanol-baited traps. We compared trap efficacy using denatured ethanol versus absolute ethanol in orchards, tree nurseries, and lumber yards in northeastern Ohio, USA, finding that absolute ethanol traps captured significantly more *X. germanus*. Analysis revealed acetone, ethanol, and methyl isobutyl ketone in the denatured ethanol, likely impacting trap efficacy. Our study underscores the importance of using pure denatured ethanol without acetone for effective monitoring, especially for *X. germanus*. Exotic xyleborines dominated trap captures across various habitats, emphasizing the need for tailored pest management strategies. Further research is warranted to explore the chemical ecology of ambrosia beetles and the influence of ethanol impurities on trap effectiveness.

## 1. Introduction

Ambrosia beetles (Coleoptera: Curculionidae: Scolytinae) are a diverse group that includes several species of serious pests in forests, lumber yards, tree nurseries, orchards, and urban greenery. Ambrosia beetles are not a clade but a polyphyletic assemblage of at least eleven different lineages within the weevil family [1]. The galleries created in wood are inoculated with spores of fungal symbionts carried in specialized cuticular structures called mycangia. Mycelia growing on the galleries’ walls are the sole source of nutrition for the beetles and their larvae [2].

Members of the ambrosia beetle tribe Xyleborini include some of the most successful insect invaders worldwide [3,4]. In addition to the cryptic lifestyle exhibited by all scolytines, the colonization of novel areas by Xyleborini is facilitated by their polyphagous habits and peculiar breeding system. Xyleborini are all haplodiploid and sib-mating, thus allowing a single fertilized female to establish a new population without any negative effects stemming from inbreeding depression [5]. Some species of invasive ambrosia beetles select healthy hosts for colonization [1], while other species preferentially infest stressed hosts [6]. Invasive ambrosia beetles pose a threat to forests [1], as well as managed habitats such as ornamental nurseries and tree fruit orchards in North America [7,8].

Within Xyleborini, the non-native members of *Xylosandrus* in N. America are among the most aggressive and damaging species [9]. *Xylosandrus germanus* (Blandford, 1894) is a xyleborine native to Eastern Asia and established in N. America [4] and Europe [10]. It is notable that in Europe, *X. germanus* is an important structural pest predominantly on dead trees in forests and logs in lumber yards [10], whereas in N. America, it attacks mostly living, albeit stressed, trees in ornamental tree nurseries and fruit tree orchards [11,12]. Another problematic xyleborine in nurseries and orchards is *Xylosandrus crassiusculus* (Motschulsky, 1866), also native to Eastern Asia and established in North and Central America, Africa, and Oceania, and introduced to Europe [7,13]. Both *X. germanus* and *X. crassiusculus* are generalists that colonize over 200 and 120 species of woody plants, respectively [14,15].

The pest status of the fruit-tree pinhole borer *Xyleborinus saxesenii* (Ratzeburg, 1837), native to Europe, Asia, and North Africa [16], and introduced over 100 years ago into N. America, is not as clear. While Rabaglia et al. [9] listed *X. saxesenii* as one of the most damaging xyleborines in NA, Ranger et al. [17] concluded that it primarily colonizes stressed, weakened or dying trees, and is not a pest of healthy trees. A much more recent xyleborine invader in NA, *Anisandrus maiche* (Stark, 1936), native to Asia, was detected for the first time in Pennsylvania in 2005, and subsequently in West Virginia and Ohio. It is uncertain if *A. maiche* poses a major threat to trees in nurseries and orchards as other invasive Xyleborini [18]; however, it has been observed attacking flood-stressed *Cornus florida* L. trees in Ohio [19], and it has been suggested that it could present a risk to ornamental and horticultural trees [7].

Key species of invasive ambrosia beetles, including *X. germanus*, *X. crassiusculus*, *X. saxesenii*, and *A. maiche*, can be monitored using ethanol-baited traps [20,21]. Ethanol is emitted from stressed, dying, or dead trees, and serves as an olfactory cue aiding beetles in locating and colonizing suitable hosts [6]. Traps baited with ethanol lures can assess beetle abundance and species composition, and are a part of attempts to develop management tactics [22].

Lures prepared using absolute ethanol are commercially available. Lures can also be handmade, and denatured ethanol has been suggested as a less expensive alternative to absolute ethanol [7,23]. Pajek et al. [24] reported that traps baited with lures emitting denatured ethanol are comparable in their effectiveness to traps baited with absolute ethanol for monitoring ambrosia beetles. Using denatured ethanol as a lure for trapping ambrosia beetles could increase the cost-effectiveness of trapping studies and citizen science efforts, since it is less expensive than absolute ethanol. However, the presence of impurities in denatured ethanol could interrupt attraction to ethanol. The objective of our current study was to compare the attractiveness of traps baited with denatured ethanol vs. absolute ethanol for capturing invasive ambrosia beetles in ornamental tree nurseries, tree fruit orchards, and lumber yards in northeastern Ohio, USA.

## 2. Materials and Methods

### 2.1. Ambrosia and Bark Beetle Trapping

Bottle traps were assembled based on Ranger et al. [7,17]. In short, a Tornado Tube^®^ (Steve Spangler Science, Englewood, CO, USA) connected the mouth of a 1 L plastic bottle to that of a 0.5 L plastic bottle. Two rectangular openings (length 11 cm, width 7 cm) were cut into the 1 L bottle, allowing the entrance of beetles. Lures were created by drilling four holes (5 mm diam.) at quarterly intervals along the perimeter of the upper portion of a 50 mL conical polypropylene centrifuge tube (Falcon™, Corning Inc., Corning, NY, USA) to allow for evaporation of ethanol. The centrifuge tubes were filled with 25 mL of absolute ethanol (95%, 190 proof, 0.805 g/mL, Decon Labs, Inc., King of Prussia, PA, USA) diluted to 70% with water (*v*:*v*) or denatured ethanol (70% ethanol, *v*:*v*; inactive ingredients—acetone, denatonium benzoate, and methyl isobutyl ketone; CVS Pharmacy, Woonsocket, RI, USA). A wire wrapped underneath the cap of the centrifuge tube was used to suspend the tube within the upper 1 L trap bottle. The mean release rate from the vials at 26 °C was 799.8 mg/day for 70% absolute ethanol and 1014.6 mg/day for 70% denatured ethanol. A mixture of water:propylene glycol (1:1; Sierra Antifreeze/Coolant; Old World Industries, Inc., Northbrook, IL, USA) was added to the 0.5 L collecting bottle as a killing agent (approximately 50 mL).

**Figure 1:**
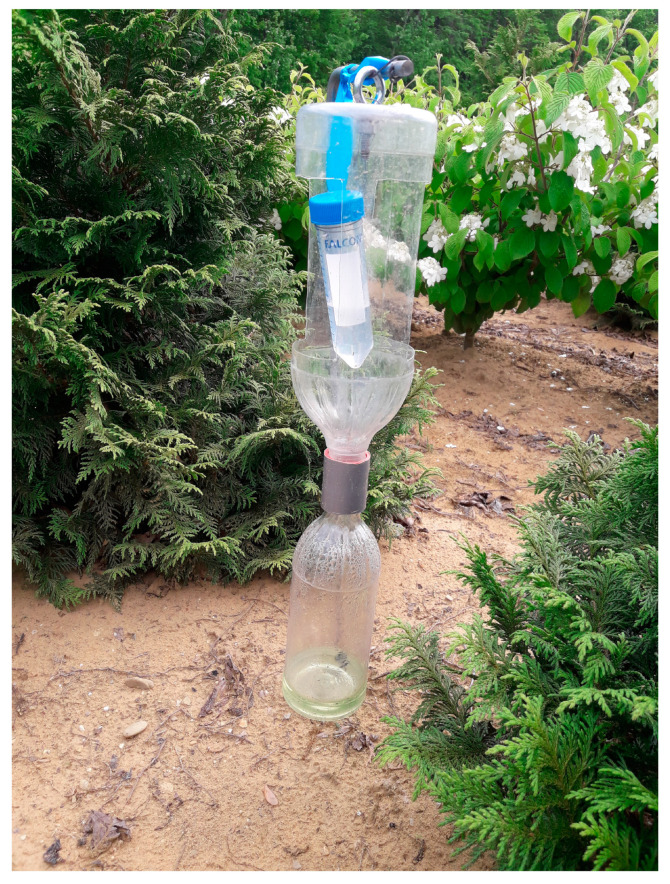
Bottle trap design used in this study placed within an ornamental tree nursery.

On 7 May 2018, we deployed a total of 36 bottle traps baited with either pure 70% ethanol or 70% denatured ethanol at nine sites representing managed habitats in northeastern Ohio, USA. Three of the sites were ornamental tree nurseries, three were fruit tree orchards, and three were lumber yards. Traps were suspended vertically 0.6 m above ground level by attaching the inverted end of the 1 L bottle to a metal rod with a wire. Four traps were deployed at each site consisting of two traps baited with 70% absolute ethanol and two baited with 70% denatured ethanol. Two of the four traps were deployed within the interior of the site 25 m from the forest edge and two traps were positioned at the forest edge neighboring the site (Figure 1).

Different treatments were placed randomly and rotated each week during the collection of specimens to rule out the effect of trap placement. Traps were placed 25 m from each other to ensure independence of sampling. The volume of ethanol within the centrifuge tube lures was replenished weekly to the 25 mL gradation. Traps were processed once per week to collect beetles over four weeks until 6 June 2018. Beetles were identified to the species level, except for *Hypothenemus* spp. and *Pityophthorus* spp., which were identified to genus level. Both genera are species-rich and morphologically difficult to distinguish [25,26].

### 2.2. Ethanol Analysis

The absolute and denatured ethanol used in our study were analyzed using solid phase microextraction–gas chromatography–mass spectrometry (SPME-GC-MS) to assess impurities. The ethanol solutions were diluted to 10% (*v*:*v*) in distilled/deionized water and 1 µL was applied to a filter paper disc within a 1.5 mL glass autosampler vial (Sigma-Aldrich, St. Louis, MO, USA) and immediately capped. After the vials were equilibrated at room temperature for 5 min, the tip of a SPME syringe was inserted through the septa cap of the GC vial. The SPME fiber (CAR-PDMS; 75 μm coating; Sigma-Aldrich) was exposed for 30 s within the vial and then retracted. Fibers were thermally desorbed for 2 min at 250 °C in the injection port of an Agilent 7890B GC (Agilent Technologies, Palo Alto, CA, USA) with an SPME liner (0.75 mm × 6.35 mm × 78.5 mm, i.d. × o.d. × length; Restek, Bellefonte, PA, USA) in splitless mode. A DB-5MS column (0.25 μm × 30 m × 0.25 mm; i.d. × length × film thickness; cross-linked/surface bonded 5% phenyl, 95% methylpolysiloxane; Agilent J&W, Santa Clara, CA, USA) was used with a temperature program of 40 °C for 2 min followed by a ramp at 15 °C/min to 200 °C. An Agilent 5977A mass spectrometer was operated in electron impact mode with a scan range of 33 to 120 amu. Five replicates of the absolute and denatured ethanol were analyzed.

### 2.3. Statistical Analyses

For the predominant species of ambrosia beetles captured in traps, count data of individual ambrosia beetle species were first pooled across the three habitat types (i.e., nursery, orchard, and lumber yard) to compare captures using absolute ethanol vs. denatured ethanol. Count data from the two absolute ethanol traps deployed at each habitat type were averaged for analysis, as were data from the two denatured ethanol traps at each habitat type. Count data between traps baited with absolute vs. denatured ethanol were compared using generalized linear models (SAS Institute). Due to non-normality, negative binomial distributions and log link functions were used to fit the models, as confirmed by values close to 1.0 for the scaled deviance (G2/df) parameter. Differences of least squares means were used for pairwise comparisons of treatment effects (α = 0.05). If a significant difference was detected between the absolute vs. denatured ethanol traps using data pooled across the three habitat types, then trap counts from the nursery, orchard, and lumber yard habitats were separately compared.

## 3. Results

### 3.1. Scolytinae Composition and Abundance

In total, 2397 specimens of 18 species of Scolytinae were captured during the trapping experiments conducted in three different artificial ecosystems (i.e., orchards, nurseries, lumber yards) in northeastern Ohio, USA. Of these, 1765 specimens were of eight species of exotic ambrosia beetles of the tribe Xyleborini, which accounted for 73.63% of all captured specimens and 44.4% of the total number of captured species. The most abundant species was *X. germanus* (749 specimens, 31.3% of all captured specimens), followed closely by *X. saxesenii* (736 specimens, 30.7% of all captured specimens) (Table 1).

### 3.2. Influence of Ethanol Grade

**Figure 2:**
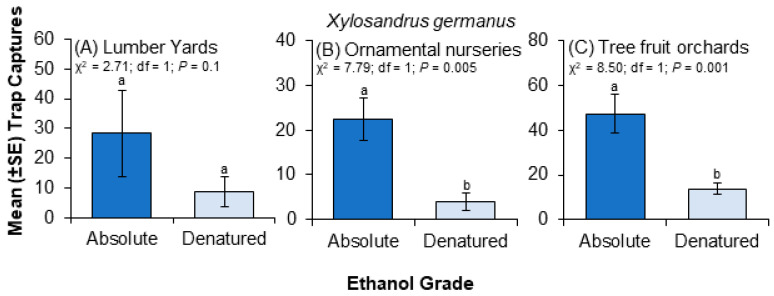
Influence of ethanol grade (i.e., absolute vs. denatured) on trap captures of *Xylosandrus germanus* at three different types of artificial ecosystems (i.e., lumber yard (**A**), nursery (**B**), orchard (**C**)). Differences at the 0.05 significance level are indicated by different letters above the vertical lines.

**Figure 3:**
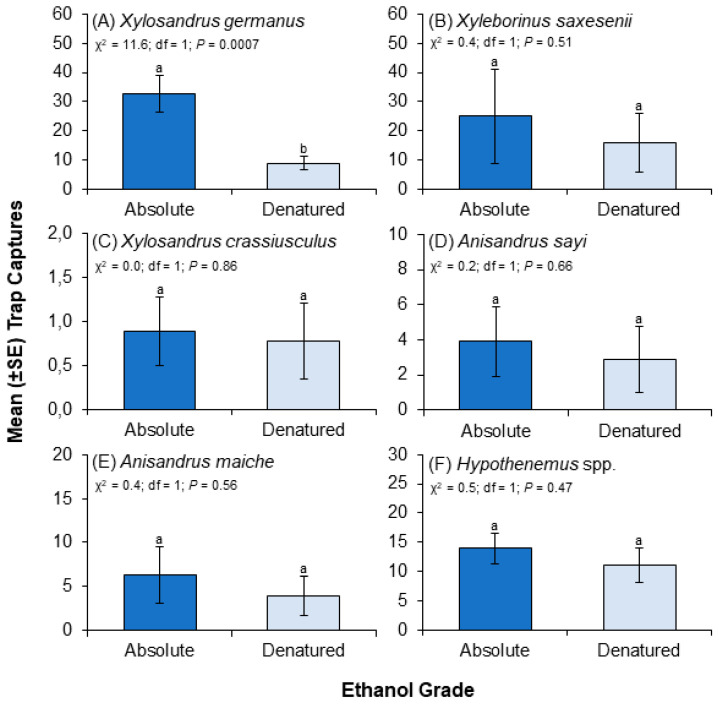
Influence of ethanol grade (i.e., absolute vs. denatured) on trap captures of selected scolytine species ((**A**) *X. germanus*, (**B**) *X. saxesenii*, (**C**) *X. crassiusculus*, (**D**) *A. sayi*, (**E**) *A. maiche*, (**F**) *Hypothenemus* spp.) in all three types of artificial ecosystems (i.e., orchard, nursery, lumber yard). Differences at the 0.05 significance level are indicated by different letters above the vertical lines.

Significantly more *X. germanus* were captured in traps baited with absolute ethanol compared to denatured ethanol when trap captures were pooled across the three habitat types (i.e., nurseries, orchards, and lumber yards). Significantly more *X. germanus* were captured using absolute ethanol vs. denatured ethanol in ornamental nurseries (Figure 2B) and tree fruit orchards (Figure 2C), but no difference was detected between the ethanol grades for traps deployed in lumber yards (Figure 2A). No significant differences were detected in trap captures of *X. saxesenii* (Figure 3B), *X. crassiusculus* (Figure 3C), *Anisandrus sayi* (Figure 3D), *A. maiche* (Figure 3E), and *Hypothenemus* spp. (Figure 3F) between traps baited with absolute vs. denatured ethanol when counts were pooled across the three habitat types.

### 3.3. Ethanol Analysis

Analysis by SPME-GC-MS of the denatured ethanol used in the trapping study detected the presence of acetone, ethanol, and methyl isobutyl ketone as primary components (Table 2). In contrast, ethanol was the only major or minor component detected in the absolute ethanol used in the trapping study.

## 4. Discussion

Almost ¾ of individual beetles (73.6%) and nearly ½ of species (44.4%) of all captured Scolytinae in our study were exotic species of the tribe Xyleborini (Table 1). Thus, non-native species were the most abundant scolytines in our three selected habitat types (i.e., nurseries, orchards, and lumber yards), which is in accordance with Gandhi et al. [27], who also investigated the semiochemical response and species composition of bark and ambrosia beetles in northeastern Ohio. *Xylosandrus germanus* and *X. saxesenii* were the dominant exotic species collected as part of our study.

Monitoring the flight activity of ambrosia beetles is important for pest management practices. Handmade lures using denatured ethanol could be a less expensive alternative to commercially available lures. Our current study found that baiting traps with denatured ethanol vs. absolute ethanol reduced trap captures of *X. germanus* within orchards and nurseries, but not within lumber yards. This is in contrast with Pajek et al. [24], who found that denatured ethanol was similarly effective at capturing some xyleborines as absolute ethanol. Acetone, denatonium benzoate, and methyl isobutyl ketone were listed as ingredients of the denatured ethanol used in our study and the presence of these compounds was confirmed using SPME-GC-MS. The presence of acetone in the denatured ethanol could have interrupted the attraction of *X. germanus* to ethanol. Indeed, Montgomery and Wargo [28] found that while ethanol-baited traps were attractive to scolytines, traps baited with acetone were not attractive. Traps baited with lures releasing acetone were not attractive to *X. germanus* or other ambrosia beetles [17]. Similar results were obtained after injecting *Magnolia virginiana* L. trees with acetone, which induced a minimal number of attacks compared to ethanol. The presence of methyl isobutyl ketone within the denatured ethanol may have also interrupted the attraction of *X. germanus* to ethanol.

Species of *Hypothenemus*, however, may possibly pose an exception. This genus includes many poorly understood species, as well as the well-studied and economically significant pest of coffee worlwide, the coffee berry borer *Hypothenemus hampei* (Ferrari, 1867) [26]. In our study, we noticed that numbers of captured *Hypothenemus* spp. specimens were not significantly different between the two ethanol treatments, with nearly half of all individuals (44.35%) captured in traps baited with denatured ethanol containing acetone (Table 1). It has been shown that females of *H. hampei* respond to unidentified volatiles obtained from coffee berry acetone extracts [29,30]. However, the role of acetone in the chemical ecology of *H. hampei* and other species of *Hypothenemus* has not been studied in detail, and our results may warrant further investigation.

In conclusion, we found that exotic xyleborines dominate in captures in ethanol-baited traps placed within ornamental tree nurseries, fruit tree orchards, lumber yards, and the interface between these artificial ecosystems and adjacent forest stands in northeastern Ohio. The denatured ethanol used in our study was not as effective in some cases as absolute ethanol, probably due to acetone content. Thus, we recommend that only denatured ethanol without acetone be used in the monitoring of *X. germanus* and other Scolytinae, which use ethanol to locate suitable hosts.

## Figures and Tables

**Table 1:** Species, mean captures by artificial ecosystem, standard error of the mean (±SE), and total captures of Scolytinae.

Species	Mean Captures by Artificial Ecosystem			Total
	Orchard	Nursery	Lumber Yard	
Native species				
* Anisandrus sayi* Hopkins	10.7 ± 4.9	28.0 ± 22.5	2.0 ± 1.1	122
* Hypothenemus* spp. Westwood	51.7 ± 22.4	75.0 ± 6.8	27.0 ± 3.5	451
* Micracis swainei* Blackman	0.0 ± 0.0	0.3 ± 0.3	0.7 ± 0.7	3
* Monarthrum fasciatum* Say	0.3 ± 0.3	0.0 ± 0.0	5.3 ± 5.3	17
* Monarthrum mali* Wood & Bright	0.0 ± 0.0	0.0 ± 0.0	6.0 ± 4.5	18
* Orthotomicus caelatus* Eichhoff	0.0 ± 0.0	0.7 ± 0.7	0.0 ± 0.0	2
* Pityophthorus* spp. Eichhoff	3.3 ± 2.8	0.3 ± 0.3	0.3 ± 0.3	12
* Phloetribus liminaris* Harris	0.6 ± 0.6	0.3 ± 0.3	0.0 ± 0.0	3
* Xyleborus ferrugineus* Fabricius	0.3 ± 0.3	0.3 ± 0.3	0.3 ± 0.3	3
* Xyleborus pubescens* Zimmermann	0.0 ± 0.0	0.0 ± 0.0	0.3 ± 0.3	1
Exotic species				
* Ambrosiophilus atratus* Eichhoff	2.3 ± 1.3	0.3 ± 0.3	1.3 ± 0.9	12
* Anisandrus maiche* Stark	30.3 ± 25.0	4.7 ± 4.2	26.0 ± 22.5	183
* Cyclorhipidion bodoanum* Reitter	2.0 ± 1.5	2.0 ± 2.0	1.0 ± 1.0	15
* Dryoxylon onohaerense* Murayama	0.0 ± 0.0	11.7 ± 11.7	0.3 ± 0.3	36
* Euwallacea validus* Eichhoff	0.3 ± 0.3	0.3 ± 0.3	0.7 ± 0.7	4
* Xyleborinus saxesenii* Ratzeburg	176.3 ± 159.9	18.7 ± 14.3	50.3 ± 21.7	736
* Xylosandrus crassiusculus* Motschulsky	4.0 ± 4.0	5.7 ± 2.9	0.3 ± 0.3	30
* Xylosandrus germanus* Blanford	122.3 ± 22.2	52.7 ± 13.5	74.7 ± 38.9	749

**Table 2:** SPME-GC-MS analysis of denatured ethanol used to assess attractiveness to ambrosia beetles during trapping studies conducted in three different artificial ecosystems.

Compound Detected	Retention Time (min)		Relative Composition (%)	
Ethanol	1.49		24.8	±2.5
Acetone	1.55		48.7	±2.1
Methyl Isobutyl Ketone	3.03		26.6	±1.4

## Data Availability

The raw data supporting the conclusions of this article will be made available by the authors on request.
